# Two Interesting Cases of Thyroid Gland Tuberculosis

**DOI:** 10.7759/cureus.68733

**Published:** 2024-09-05

**Authors:** Madhu Sowmitha Pachipala, Senthil Kumar Aiyappan

**Affiliations:** 1 Department of Radiodiagnosis, SRM Medical College Hospital and Research Centre, Sri Ramaswamy Memorial Institute of Science and Technology (SRMIST), Chengalpattu, IND

**Keywords:** computed tomography, fnac, lymph nodes, tb-pcr, ultrasound

## Abstract

Tuberculosis involving the thyroid gland is an exceptionally rare condition with varied clinical presentations, often leading to diagnostic challenges. We report two cases: a 9-year-old male with necrotic tuberculous cervical lymphadenopathy secondarily involving the thyroid gland, and a 40-year-old male with disseminated tuberculosis affecting multiple organ systems, including the thyroid gland. Both cases presented with swelling over the neck region and were evaluated using ultrasonography and contrast-enhanced computed tomography (CECT), which revealed characteristic imaging findings of thyroid involvement. Fine-needle aspiration cytology (FNAC) and TB-PCR (polymerase chain reaction for Mycobacterium tuberculosis) of the aspirate confirmed the diagnosis of tuberculosis. Early identification and medical management with anti-tubercular therapy led to successful treatment, thereby avoiding unnecessary surgical interventions. These cases signify the importance of considering thyroid tuberculosis in the differential diagnosis of thyroid lesions, especially in endemic regions, and highlight the role of imaging and FNAC in establishing a prompt diagnosis.

## Introduction

Tuberculosis involving the thyroid is a rare entity even in countries like India where it is an endemic disease [1]. Symptoms of thyroid tuberculosis are nonspecific and often lead to diagnostic challenges. Here, we present two cases of tuberculosis involving the thyroid gland, the first case being necrotic tuberculous cervical lymphadenopathy with abscess formation, secondarily involving the left lobe of the thyroid, and the second case being disseminated tuberculosis with multi-system involvement along with thyroid involvement. We highlight the importance of considering thyroid tuberculosis in the differential diagnosis of thyroid lesions, especially in endemic regions, and how the imaging findings and fine-needle aspiration cytology (FNAC) have played an important role in establishing a prompt diagnosis, which led to the successful management of the cases.

## Case presentation

Case 1

**Figure 1:**
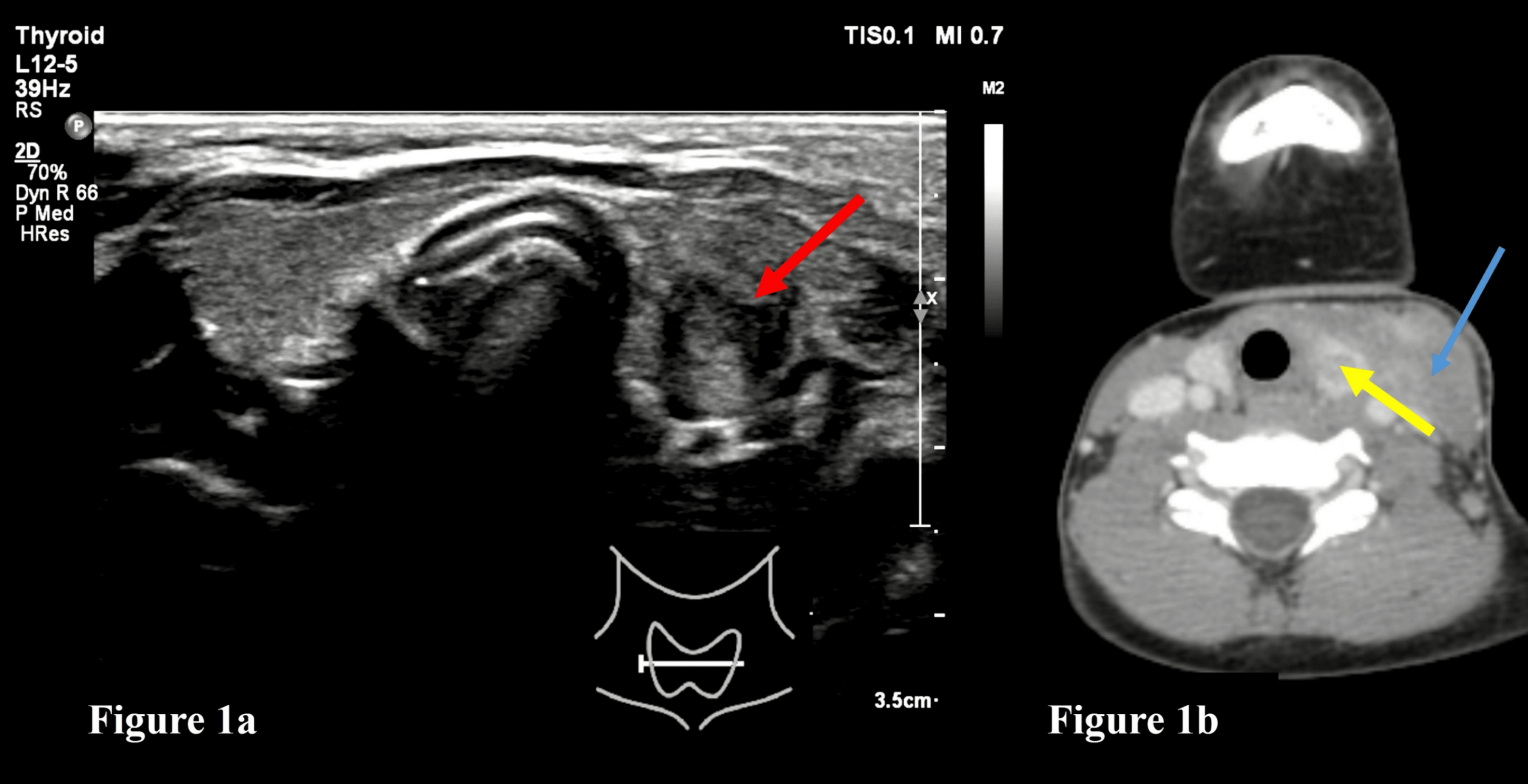
Ultrasound and CECT of the neck a: Ultrasonography of the neck region (transverse section) shows a heterogenous hypoechoic lesion involving the left lobe of the thyroid (red arrow); b: Axial section of contrast-enhanced computed tomography (CECT) of the neck shows a conglomerate lymph nodal mass on the left side of the neck (blue arrow), with extension into the left lobe of the thyroid (yellow arrow).

A nine-year-old male child presented to the pediatrics outpatient department with complaints of swelling in the left side of the neck for four months, which increased in size during the previous two days. There were no complaints of fever, cough, cold, vomiting, loose stools, abdominal pain, or breathing difficulty. There was no history of contact of tuberculosis in the family. There was no significant antenatal, birth, or neonatal history. The developmental history of the child was normal, and immunization was up to date. The general physical examination of the child was normal. On local examination, there was a swelling of size 3 x 3 x 1 cm on the left side of the neck, anterior to the sternocleidomastoid muscle, which was tender on palpation. The swelling had a smooth surface and was firm in consistency. The blood investigations, including complete blood count (CBC), liver function tests, renal function tests, and the thyroid hormone profile were normal. Cartridge-based nucleic acid amplification test (CB-NAAT) and acid-fast bacilli (AFB) from the gastric aspirate were negative. The Mantoux test showed no induration. The child was admitted to the hospital and was evaluated for cervical lymphadenopathy to rule out lymphoma/tuberculous lymphadenitis. Chest radiograph showed no features of tuberculosis. Ultrasonography of the neck region revealed a heterogeneously hypoechoic lesion measuring 32 x 27 x 15 mm with anechoic areas within, noted involving the left lobe of the thyroid. The lesion was noted anterior and medial to the sternocleidomastoid muscle with involvement of the sternomastoid. In addition, necrotic lymph nodes were noted in the level II, III, and IV stations of the neck on the left side. The lesion was seen extending into the left lobe of the thyroid gland (Figure 1a). In addition, a few other enlarged bilateral upper deep cervical lymph nodes were noted. Contrast-enhanced computed tomography (CECT) neck was done to further characterize the lesion, which showed a large, solid, enhancing lesion on the left side of the neck involving levels II, III, and IV, showing non-enhancing necrotic areas within, with extension into the left lobe of the thyroid gland. The lesion was suggestive of a necrotic conglomerate lymph nodal mass on the left side of the neck and associated involvement of the left lobe of the thyroid gland (Figure 1b).

Fine-needle aspiration cytology (FNAC) of the thyroid gland and lymph nodal mass was done, which revealed epithelioid granulomatous inflammation with suppuration, pointing toward tuberculous etiology. TB-PCR (polymerase chain reaction for Mycobacterium tuberculosis) of the FNAC aspirate was positive. The patient was subjected to the incision biopsy for the same, which revealed fibrocollagenous and fatty tissue with sheets of neutrophils, lymphocytes, plasma cells, and proliferating capillaries with infiltration into adjacent fatty tissue indicating acute-on-chronic inflammation with abscess formation. The child was started on anti-tubercular therapy with two months of intensive phase (daily dosage of isoniazid, pyrazinamide, rifampicin, and ethambutol - HRZE) followed by four months of continuous phase (thrice weekly dosage of isoniazid, rifampicin, and ethambutol - HRE). The patient showed complete resolution of symptoms after a six-month follow-up.

Case 2

**Figure 2:**
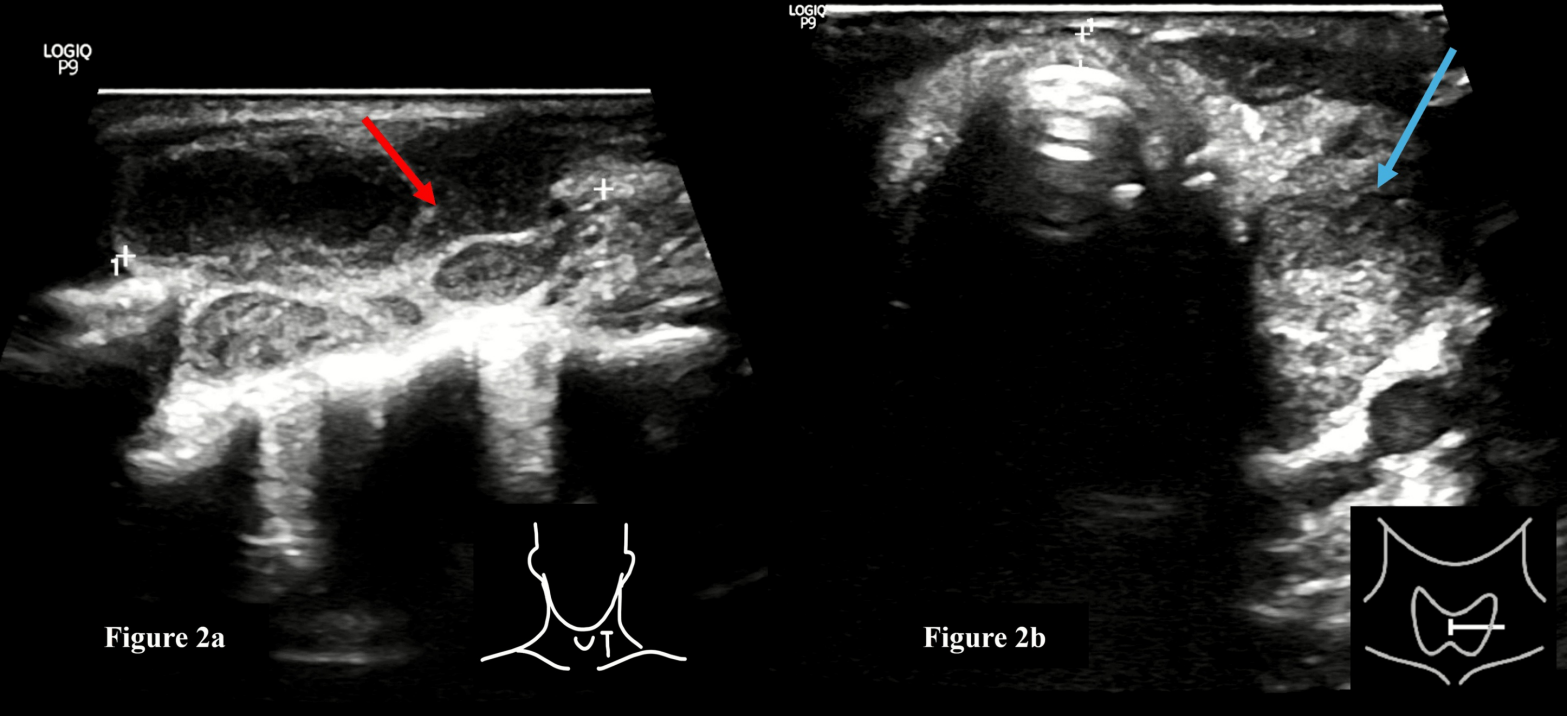
Ultrasonography of the neck a: Ultrasonography of the neck region on the left side (sagittal section) shows multiple conglomerate necrotic lymph nodes (red arrow); b: Ultrasonography of the neck region (transverse section) shows a well-defined, thick-walled cystic area with echogenic content (blue arrow) with thick internal septations noted involving the left lobe of the thyroid.

A 40-year-old male presented with complaints of generalized tiredness, cough with expectoration for the past two months, non-foul-smelling mucoid sputum, recent weight loss of 5 kilograms in the past one month, and evening rise of temperature for the past two weeks. The patient had no complaints of shortness of breath, chest pain, hemoptysis, and vomiting. The general and systemic examinations were unremarkable. On local examination, multiple enlarged cervical lymph nodes were noted in the left upper, mid, and lower deep cervical region and the right lower deep cervical region. The routine blood investigations revealed that the patient was anemic with hemoglobin 7.6 gm/dl (Normal: 13-17 gm/dl). The total leukocyte count was within normal limits. The rest of the blood investigations and urine analysis were normal. A CB-NAAT test of sputum revealed the presence of Mycobacterium tuberculosis with indeterminate rifampicin resistance. Given neck swelling, the patient was subjected to ultrasonography (USG) neck, which revealed multiple necrotic lymph nodes in levels II, III, IV, and V on the left side and levels II, IV, and V on the right side (Figure 2a). In addition to that, a relatively well-defined, thick-walled cystic area measuring ~ 3.8 x 2.4 x 1.6 cm, volume 10.5 ml, with thick internal septations and echogenic content within, was noted involving the left lobe of the thyroid gland (Figure 2b).

**Figure 3:**
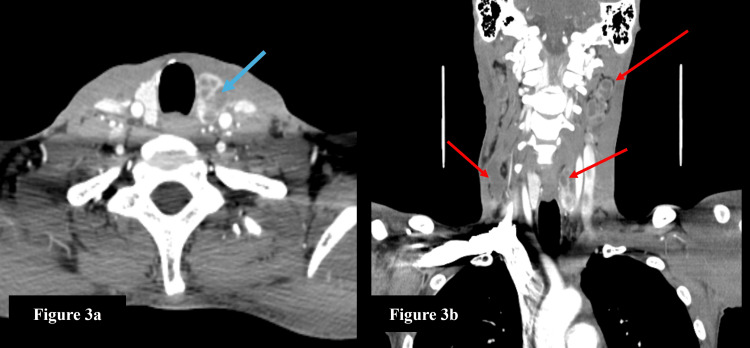
Contrast-enhanced CT of the neck a: The axial section of contrast-enhanced CT of the neck shows a well-defined multiloculated collection with enhancing septations within the left lobe of the thyroid gland (blue arrow); b: The coronal section of contrast-enhanced CT of the neck shows multiple, enlarged, and conglomerate cervical lymph nodes (red arrows) with non-enhancing hypodense necrotic areas on both sides.

On further evaluation, the CECT neck revealed a relatively well-defined multiloculated fluid density collection measuring ~ 3.5 x 2.2 x 1.4 cm (CC x AP x TR) with few enhancing septations within seen involving the left lobe of the thyroid gland (Figure 3a). Multiple enlarged conglomerate cervical lymph nodes with non-enhancing hypodense areas, suggestive of necrosis noted in levels IIb, IV, and Vb and supraclavicular stations on the right side and levels IIa, IIb, III, IV, and Va on the left side, with the largest measuring ~ 3.1 x 2.5 x 1.5 cm at level III on the left (Figure 3b).

**Figure 4:**
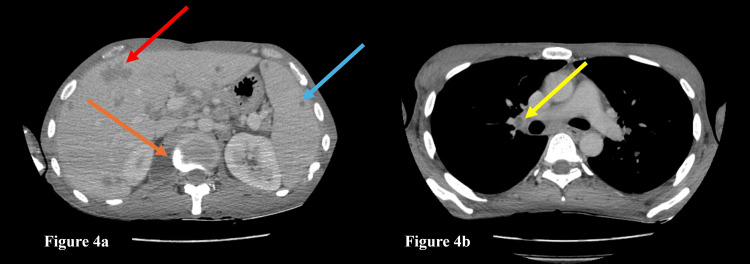
Contrast-enhanced CT (CECT) of the chest and abdomen a: The axial section of the CECT abdomen shows multiple hepatic lesions (red arrow), splenic lesions (blue arrow), and a paravertebral abscess (orange arrow); b: The axial section of the CECT chest shows a necrotic right hilar lymph node (yellow arrow).

**Figure 5:**
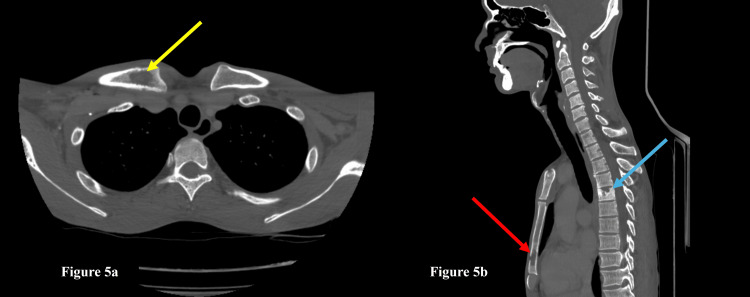
CT chest and abdomen in bone window reconstruction a: The axial section of the CT chest in bone window reconstruction shows a lytic lesion in the right clavicle (yellow arrow); b: The sagittal section of the CT chest in bone window reconstruction shows lytic lesions in the sternum (red arrow) and D5 vertebral body destruction (blue arrow).

In the same setting, a CECT chest and upper abdomen was also done, which showed the involvement of the chest in the form of necrotic mediastinal lymph nodes, a paravertebral abscess on the right side extending from the D11-L2 levels, multiple liver and spleen lesions, and lytic areas in the right clavicle, sternum, and D5 vertebral body, suggestive of disseminated tuberculosis (Figures 4, 5).

FNAC of the thyroid gland revealed necrotizing epithelioid granulomatous inflammation with focal suppurative change. TB-PCR of the aspirated sample was positive. The patient was started on anti-tubercular therapy with two months of intensive phase (daily dosage of isoniazid, pyrazinamide, rifampicin, and ethambutol - HRZE) followed by 12 months of the continuous phase (thrice weekly dosage of isoniazid, rifampicin, and ethambutol - HRE) for disseminated tuberculosis.

## Discussion

The common sites of extrapulmonary tuberculosis are the pleura, lymph nodes, skeletal system, and central nervous system [1,2]. Involvement of the thyroid gland is very rare, seen only in 0.1-0.4% cases, and this rarity can be attributed to high iodine levels, antibacterial action of colloid, high vascular supply, well-developed capsule, and possible antitubercular role of thyroid hormones [3,4]. The pathway to infection of the thyroid gland can be through hematogenous or lymphatic dissemination from the remote focus of infection [5]. It has a variable presentation from being asymptomatic to causing variable pathologies like multiple lesions diffusely involving the gland, goiter with caseating necrosis, cold abscess within the gland, acute abscess formation, and chronic fibrosing tuberculosis. Patients can be euthyroid in the initial stages of involvement but in later stages, hypo/hyperthyroidism could set in [5,6].

The clinical presentation is variable, the commonest one being a solitary nodule, but it can also present as an abscess or a fast-growing goiter. In rare cases, it can present with dyspnoea or dysphagia due to the involvement of adjacent structures [7,8]. FNAC may diagnose thyroid tuberculosis in up to 73% of cases [9]. FNAC shows epithelioid granulomas with Langhans’ giant cells and Caseation necrosis [10,11]. TB-PCR is a potentially useful method of detection of Mycobacterium tuberculosis DNA from FNAC of thyroid lesions and provides an alternative for rapid diagnosis of thyroid tuberculosis in AFB-negative cases. TB-PCR increases the sensitivity of cytological examination, and it is extremely useful when culture results are negative or when differentiation from other forms of granulomatous thyroiditis is difficult [12].

Talapa et al. reported a case of thyroid tuberculosis wherein the patient presented with multiple palpable nodules in both lobes of the thyroid along with multiple matted cervical lymph nodes. On subsequent follow-up, the patient had a life-threatening pericardial effusion secondary to hypothyroidism [13]. In our cases, the patients presented with similar complaints of palpable bilateral cervical lymph nodes and left lobe of thyroid involvement. However, in the current report, both patients were euthyroid. Dv K et al. reported a case of disseminated tuberculosis in a patient with a history of tuberculosis who had been a treatment defaulter and presented with disseminated tuberculosis with brain and thyroid gland involvement. The patient is a treatment defaulter and the thyroid gland involvement is similar to the second case presented in the current report. The author attributed the thyroid gland involvement to a lack of compliance with treatment and inadequate doses of antitubercular therapy [14].

Rojo-Abecia M et al. reported a case of primary thyroid tuberculosis in which the patient presented with a nodule in the left lobe of the thyroid, which showed irregular borders and mimicked thyroid malignancy for which the patient underwent hemithyroidectomy [15]. The postoperative specimen revealed granulomatous thyroiditis with non-necrotizing epithelial granulomas and acid-fast bacilli were demonstrated using Ziehl-Nielsen staining. Chacko A et al. reported a case of thyroid tuberculosis that was diagnosed as a malignant thyroid nodule, wherein the patient presented with a palpable thyroid nodule on the right side with subclinical hypothyroidism for which total thyroidectomy was done. The postoperative specimen revealed multiple discrete and coalescent granulomas composed of Langhans giant cells, epitheloid histiocytes, and lymphocytes, and the Ziehl Neilsen stain revealed acid-fast bacilli [16].

In the above-discussed cases, the imaging findings of tuberculosis involving the thyroid gland included sonographic evaluation and evaluation using computed tomography, which has a significant role in identifying the lesion and establishing the final diagnosis.

In the current cases, the patients were managed conservatively due to the detection of tuberculosis of the thyroid gland on imaging, granulomatous inflammatory changes on FNAC, and TB-PCR being positive for the FNAC aspirate had allowed starting the patients on an appropriate antitubercular regimen that avoided the invasive surgical option and life-long maintenance thyroid therapy.

## Conclusions

In conclusion, tuberculosis of the thyroid gland is a rare manifestation of a common endemic disease with varied clinical manifestations, and it should be considered an important differential diagnosis while evaluating neck swellings and thyroid nodules. Imaging modalities like ultrasonography and contrast-enhanced computed tomography can be used in diagnosing the condition even in areas where there is no access to high-tech diagnostics like TB-PCR.
